# Neuro Pathology and Psychological Medicine

**Published:** 1874-04

**Authors:** Walter Hay

**Affiliations:** Adjunct Professor Theory and Practice, Lecturer on Diseases of the Brain and Nervous System, in Rush Medical College


ARTICLE I.—*Neuro Pathology and Psychological Medicine.* By WALTER HAY, M.D., Adjunct Professor Theory and Practice, Lecturer on Diseases of the Brain and Nervous System, in Rush Medical College.

1. *Mendel on Typhus and Diseases of the Mind.* (Berliner Medizinischer Wochenschrift, Sept. 23, 1873. London Medical Record, Nov. 19, 1873.)

2. *Eulenberg on Lesions of Innervation apparently due to Ill-Usage.* (Berliner Klinische Wochenschrift, Sept. 23. London Medical Record, November 19, 1873.)

3. *Vertigo ab Aure læsa,* La Maladie de Meniere Clinique Medicale. Hospice de la Salpetriere. M. CHARCOT.

4. *Recherches Experimentale sur la Physiologie et la Pathologie Cerebrale.* Par le Docteur DAVID FERRIER, Professeur de Medecine Legale au King's College de Londres: Le Progres Medicale.

5. *Accommodation for the Insane on the Cottage Plan.* By WINTHROP B. HALLOCK, Assistant Physician to the Connecticut Hospital for the Insane. (New York Medical Journal.)

1. Dr. Mendel considers the relations between typhus fever and insanity from three points: (1.) The differential diagnosis; (2.) Typhus as a cause of insanity; and (3.) The influence of typhus upon existing brain disease.

(1.) The diagnosis must be established by the thermometer; the characteristic temperature—curves of typhus—rising in the morning from 37° to 38.5°, and in the evening from 40° to 40.7° (cent.)—do not appear in brain diseases.

(2.) Typhus, more frequently than other acute diseases, causes insanity, in consequence of the psychical and special organic changes accompanying it. The cases generally recover, although hallucinations may persist after the re-establishment of normal temperature. The brain symptoms occurring at the close of an attack of typhus are more urgent, and may take the form of the transitory delirium of inanition or collapse, or that of gradually developing mental disease, ending frequently in destructive lesion of the brain and consequent dementia.

The proportion of violent cases of insanity thus originating is estimated, variously, by Nasse, Schlager and Jacobi, at 2.3, 4.4 and 12.3 per cent. respectively.

(3.) Mental disease has occasionally been cured by an intercurrent attack of typhus. Bach saw ten out of eleven, and Schlager six out of eleven cases, so recover; apparently resulting from anæmia occasioned by a diminution in the circulation in the membranes of the brain. These recoveries, however, are generally temporary.

2. Dr. Eulenberg exhibited to the Hufeland Association a child eleven years old, who had been maltreated at school by blows on the head, plucking out the hair, etc., the result being epileptic vertigo, great pain in the occiput, hyperæsthesia of the nerves of sensation, etc. There were bald spots upon the scalp, which after tonic treatment and shaving, were replaced by snow-white hair, the whiteness depending on absence of pigment. Moritz Kohn speaks of alopecia areata as a trophic neurosis, as shown by its heredity, its sudden appearance and disappearance, its extension peripherally, and its connection with other nerve lesions.

Wilson saw a case where neuralgia of the head preceded the area. Wyss saw one in a boy who was taking arsenic; but on stoppage of the medicine the area vanished. Cusp, Todd, Anstie, and others, have noticed alopecia in nervous diseases. Eulenberg reports a case of right-sided supra-orbital neuralgia, with the eyebrow white over the supra-orbital foramen, and a corresponding streak of hair on the head whitened.

3. M. Charcot, the lecturer, calls especial attention to certain symptoms which while differing from those usually present in this disease, he regards as pathognomonic. These are, first, the intermittent or rather the paroxysmal character of the vertiginous attacks, their anticipation by a loud whistling (like that of a locomotive) noise in the ear, and their sudden cessation. Triquet, Saissy, Viricel, Trousseau, and Meniere, have all associated the vertigo with noises in one or both ears; they have referred to them as continuous. Charcot insists that the vertigo manifests paroxysmal exacerbations, invariably preceded by the loud sounds, which cease with its access in recent cases; these troublesome symptoms sooner or later being relieved by the supervention of deafness.

Another symptom upon which the lecturer dwells with emphasis, is the sensation (subjective wholly) of rotation, either upon a transverse axis (trapeze movement) or upon a vertical one. These subjective sensations frequently result in the fall of the patient.

He suggests, moreover, as the results of his own experience, the application of the actual cautery to the mastoid process.

4. This new series of experiments to determine the functions of different portions of the brain, confirms, for the most part, those already performed. Thus, for example, irritation of the middle external convolution, behind the furrow limiting the frontal region, induces elevation and retraction of the right ear, and elevation of the lower eyelid. A little in front of this point, irritation induces a drawing upward of the right cheek, forced contraction of the eyelids, and especially depression downward and outward of the right eye.

Irritation of the gyrus sigmoid behind the crucial furrow induces elevation of the right shoulder, with a slight movement—adduction—of the right fore paw, (of the cat), followed by choreic movements of the same parts during one or two minutes, being simply rapid repetitions of the original movements.

Irritation of the parietal regions induced rotation of the head. Irritation of the recurved portions of the external convolutions induced no movements, but extorted cries of pain.

Irritation of the anterior extremities of the infra- and supra-sylvian frontal convolutions induced the opening of the mouth, its seizure with clonic convulsions, and the projection direct forward of the tongue, the animal crying with anger and pain, and agitating his tail. Movements made so distinctly and so frequently indicated clearly the region in which they were centralized.

These movements were reproduced exactly, after the induction of profound stupor by chloroform, though without the cries.

Irritation of the posterior region of the fissure of Sylvius induced instant and violent closing of the jaws, which could thus be opened and closed alternately by irritation of the first or second region.

One of the electrodes being placed at the anterior extremity of the external superior convolution, and the other at the orbital extremity of the last frontal, and of the supra-sylvian convolutions, the head was thrown back, the jaws opened convulsively to their whole extent, and the tongue moved as before.

Similar experiments resulted thus :

(1.) The electrodes applied to the sigmoid gyrus of the frontal division of the external frontal convolution, induced *adduction of the fore paw from the right side.*

(2.) The electrodes moved a little forward upon the same gyrus, induced *elevation of the right shoulder*, *adduction of the right paw, with extension of the great toes. The hind paw of the right side was also beni and drawn forward.* The cessation of the stimulations, after several repetitions, was followed by a unilateral epileptic attack, characterized by spasms of the right eyelids, elevations of the right shoulder, and convulsive erections of the tail.

(3.) The electrodes at the extremity of the horizontal division of the crucial furrow, induced *rotation of the head toward the right shoulder.*

(4.) Electrization of a point upon the anterior limb of the sigmoid gyrus of the superior external convolution, induced immediately elevation of the right eyebrow and eyelid.

The intensity of the current was only such as permitted the electrodes to be borne at the tip of the tongue without pain.

(5.) Electrization of the posterior extremity of the frontal division of the middle external convolution, induced forcible closure of the right eye, and attraction of the head to the right.

(6.) Electrization of the middle of the same convolution, induced *results similar to the last.*

(7.) Electrodes being advanced a little upon the same convolution, *induced same effects.*

(8.) Applied to posterior extremity of the frontal division of the external inferior convolution, *with same result.*

(9.) Applied to superior external convolution, behind the sigmoid gyrus, *the tail was first moved from side to side, then elevated, and remained rigid. Contractions of the external muscles of the right thigh occurred.*

Here ensued an epileptic attack, with convulsions limited to the muscles involved in the experiments.

(10.) Electrization of the external superior convolution, at a point behind the caudal nervous centre, elicited only outcries of pain.

The analysis of these observations will be continued in a future number of the Journal.

5. Dr. Hallock objects, with good reason, to the accumulation of large numbers of insane patients in single buildings, but urges the advantages of providing detached buildings of small size—cottages, so called—to accommodate thirty patients each, under the care of two attendants.

The advantages are asserted to be, diminished cost of construction and also of maintenance, whereby provision could be made for a much larger number of insane than by the present costly system; and this is a strong point in the Doctor's argument—the proportion of insane being estimated to be one in 1,700 annually, of which number one-fourth need hospital accommodation (this estimate seems very small.)

Another advantage claimed is, economy of space and labor. All these, of course, are important, and yet entirely subsidiary to the improvement in hygiene, in facility of management, and in prospect of recovery thereby secured. The plan is apparently an effort in the right direction, and, while liable to objections, has sufficient advantages to entitle it to a fair trial, more especially as it migh be made without essential alteration in the annual estimates for these charities, or in the personnel of their administration.

The ordinary mode of consigning diseased brains to the almost exclusive association of other diseased brains, has in it an element of absurdity, when their curative treatment is kept in view; and while removal from their accustomed surroundings, and the supervision of expert care is essential to the curative treatment of the insane, it seems that we have by no means accomplished the best for them when we shall have consigned them to a large asylum for lunatics.

